# Response to letter to the editor

**DOI:** 10.1186/s13012-020-01010-1

**Published:** 2020-07-02

**Authors:** Rosmin Esmail, Heather M. Hanson, Jayna Holroyd-Leduc, Sage Brown, Lisa Strifler, Sharon E. Straus, Daniel J. Niven, Fiona M. Clement

**Affiliations:** 1 Department of Community Health Sciences, Cumming School of Medicine, University of Calgary, 3D14A Teaching and Wellness Building, 3280 Hospital Drive NW, Calgary, Alberta T2N 4Z6 Canada; 2 Alberta Health Services, Calgary, Alberta Canada; 3 O’Brien Institute for Public Health, University of Calgary, Calgary, Alberta Canada; 4 Department of Medicine, Cumming School of Medicine, University of Calgary, Calgary, Canada; 5 Hotchkiss Brain Institute, University of Calgary, Calgary, Alberta Canada; 6 Health Technology Assessment Unit, University of Calgary, Calgary, Alberta Canada; 7 Li Ka Shing Knowledge Institute, St. Michael’s Hospital, Toronto, Ontario Canada; 8 Institute of Health Policy Management and Evaluation, University of Toronto, Toronto, Ontario Canada; 9 Department of Medicine, University of Toronto, Toronto, Ontario Canada; 10 Department of Critical Care Medicine, CummingSchool of Medicine, University of Calgary, Calgary, Alberta Canada

Dear Editor,

We would like to thank you for the opportunity to respond to Dr. Glasgow’s letter and clarify issues regarding our scoping review. We also want to thank Dr. Glasgow and his colleagues for taking an interest in our publication and expressing their concerns.

The focus of our scoping review was to provide clarity through the categorization of knowledge translation (KT) theories, models, and frameworks (TMFs) to help navigate the sometimes confusing and challenging field [1]. In the scoping review, we used the approach categorization definitions provided by Nilsen to categorize each original KT TMF [2]; within this framework, RE-AIM was categorized and described as an evaluation framework; and thus, following the protocol outlined in our paper, we have not mischaracterized RE-AIM. As we and Nilsen note, these categorizations are meant to be a guideline; the distinctions among the categories are imprecise and these categories are not always recognized as separate types of approaches in the literature [2]. However, this categorization provides a starting point for users attempting to search for and select KT TMFs.

We appreciate the further clarification that has been provided by Dr. Glasgow and colleagues as developers of RE-AIM on its application as a qualitative tool, its dimensions, time intervals, and how these have evolved over time with references to the subsequent publications, presentations, and the RE-AIM website. Unfortunately, the 20-year review paper on RE-AIM was published in March 2019 after the scoping review search strategy and was not captured in the search [3]. Further, the Practical, Robust, Implementation and Sustainability Model (PRISM) was identified in the scoping review by Strifler et al. but did not fit the definition of “full-spectrum” [4]. It was therefore not included in the scoping review.

We agree that it is important to consider any subsequent iterations on KT TMFs. Papers citing further refinements to KT TMFs were captured within the limits of our search strategy and included in the relevant categories of the scoping review. To that point, the scoping review does state that there may be more updated versions of KT TMFs or variations of KT TMFs to contemplate and select from. This comment does address the issue raised by Glasgow and colleagues to ensure that readers retrieve and evaluate any subsequent papers on KT TMFs since their original publication.

We share similar goals with Dr. Glasgow and colleagues in the effort to provide further clarity to KT TMFs and their evolvement over time. Dr. Glasgow and colleagues themselves have indicated that it is not possible to review the entire literature on each KT TMF. Given the depth and breadth of KT TMFs that are available to users, it may be useful to develop a “living” repository/catalog of KT TMFs that provide the seminal paper, subsequent iterations of the KT TMF, and papers that cite its application. We invite the Implementation Science community to consider the development of such a resource.

Sincerely,

Rosmin Esmail, Heather M Hanson, Jayna Holroyd-Leduc, Sage Brown, Lisa Strifler, Sharon E Straus, Daniel J. Niven and Fiona M. Clement.
